# Supplementary material for: Neuromorphic detection and cooling of microparticles in arrays
Source: Nat Commun. 2025 Nov 27;16:10658. doi: 10.1038/s41467-025-65677-0 (PMC12660834; doi:10.1038/s41467-025-65677-0)
Supplement: Supplementary file 1 — Supplementary Information [file 41467_2025_65677_MOESM1_ESM.pdf]

Supplementary Material: Neuromorphic detection and cooling of microparticles in arrays

Yugang Ren*[1], Benjamin Siegel[2], Ronghao Yin[1], Qiongyuan Wu[1], Jonathan Pritchett[1], Muddassar Rashid[1], and James Millen†[1,3]

[1]Department of Physics, King's College London, Strand, London, WC2R 2LS, United Kingdom
[2]Wright Laboratory, Department of Physics, Yale University, New Haven,Connecticut, 06520, USA
[3]London Centre for Nanotechnology, Department of Physics, King's College London, Strand, London, WC2R 2LS, United Kingdom

(September 16, 2025)

In this Supplemental Material we provide technical details on the experiment and data analysis, including: S1 Coordinate systems; S2 Mode identification for multiple particles; S3 Extended information for the four-particle dataset; S4 Latency in the feedback loop; S5 Power consumption of the event based camera; S6 Bath temperatures T_0 for all particles; S7 Cooling limit and the noise squashing; S8 Scalability of multi-mode cooling.

S1 Coordinate systems

Our setup has two coordinate systems: the Paul trap coordinates $\{x, y, z\}$, and the camera coordinates $\{y', z'\}$, as shown in Supplementary Figure 1 and Fig. 1 of the manuscript. The Paul trap coordinates define the oscillation axes of the levitated particles. The x-axis is defined along the diagonal between the two trapping electrodes held at a DC voltage (see Methods in the main manuscript), the y-axis along the diagonal between the two trapping electrodes with an AC voltage, and the z-axis along the axis parallel to the endcap electrodes. In the camera frame, the trap x- and y-axes are projected onto the camera's y'-axis, and the camera's z'-axis is parallel to the z-axis. Therefore, we can capture the 3D motion of the levitated particles with the camera's 2D image.

*yugang.ren@kcl.ac.uk
†james.millen@kcl.ac.uk

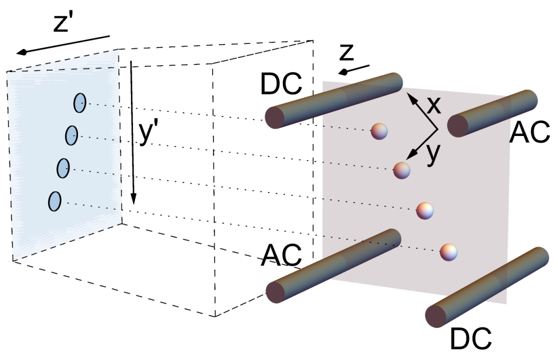

Supplementary Figure 1: Paul trap and camera coordinate systems. The Paul trap coordinates defines the axes of oscillation of the levitated particles. The x- and y-axes are along the diagonals perpendicular to DC and AC electrodes, and the z-axis is parallel to the electrodes. The Paul trap x- and y-axes make a 45° projection onto the camera y'-axis. The Paul trap z- axis is parallel to the camera z'-axis. An example of the projection of four trapped microparticles is illustrated [cf. Fig. 2 of the manuscript].

S2 Mode identification for multiple particles

Here we explain the method of identifying modes when we levitate multiple particles in an array, using a two-particle case study. We trap two charged microparticles separated by 570 µm, with the separation controlled by Paul trap voltages. Via neuromorphic detection, we calculate the power spectral densities (PSDs) of the two particles, as shown in Supplementary Figure 2(a). We identify six modes in total. To distinguish these modes, we resonantly drive the Paul trap using a sinusoidal voltage applied to the control endcap electrode, at each of the six frequencies sequentially, and observe the response of the particles using a CMOS camera, as shown in Supplementary Figure 2(b). For the upper particle the centre-of-mass motion frequencies are with $\omega_{\mathrm{x1}} = 22.2\,\mathrm{Hz}$, $\omega_{\mathrm{y1}} = 59.9\,\mathrm{Hz}$, and $\omega_{\mathrm{z1}} = 35.6\,\mathrm{Hz}$ and for the lower particle the centre-of-mass motion frequencies are $\omega_{\mathrm{x2}} = 18.2\,\mathrm{Hz}$, $\omega_{\mathrm{y2}} = 88.3\,\mathrm{Hz}$, and $\omega_{\mathrm{z2}} = 97.0\,\mathrm{Hz}$.

To identify collective modes due to interactions between the charged particles, we calculate the cross-spectral density (CSD), which picks-out only the correlated spectral components [1]. As shown in Supplementary Figure 3(a), none of the six modes exhibit significant coupling, since the separation between the particles (570 µm) is too large.

When the particles are brought into close proximity, we do observe interactions between them. We trap another two particles and reduce the separation to approximately 150 µm. We again observe six modes: 33.7 Hz and 58.1 Hz along the x-axis, 85.2 Hz and 150.9 Hz along the y-axis, and 89.4 Hz and 98.2 Hz along the z-axis. A CSD analysis in this case is given in Supplementary Figure 3(b), from which we can observe that both x-axis modes and both z-axis modes are correlated, whilst the y-axis modes appear to be uncoupled. This analysis is extended to the four particles in the manuscript below.

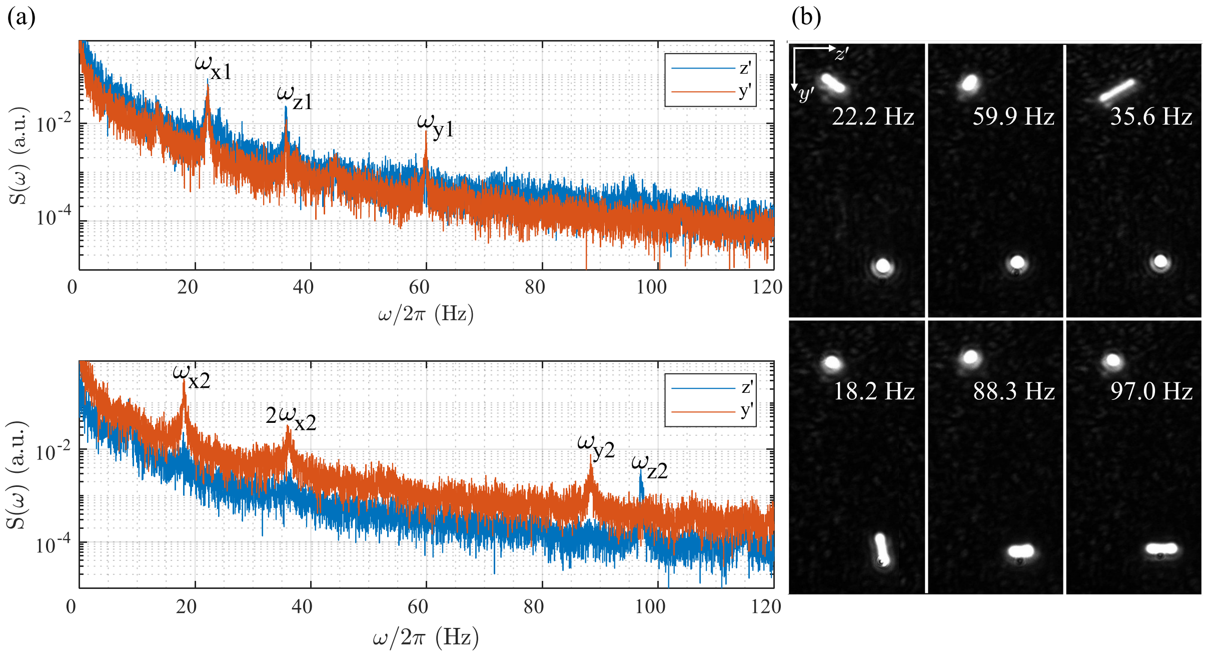

Supplementary Figure 2: Mode identification for multiple levitated particles. (a) PSDs of two levitated microparticles separated by 570 µm. The upper (lower) figure shows the PSDs along the y'- and z'-axes for particle 1 (2) respectively. **(b)** A CMOS camera view as the particles are sequentially resonantly excited by a sinusoidal voltage at each of the six frequencies seen in (a).

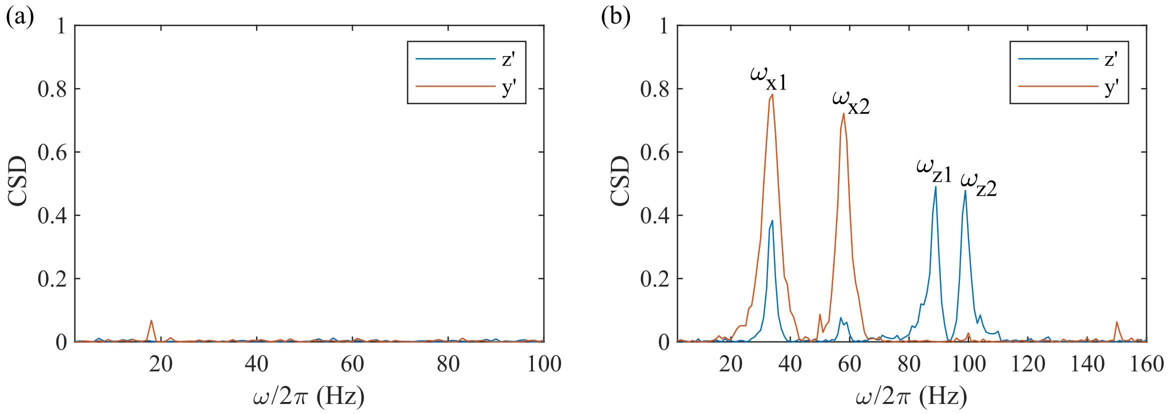

Supplementary Figure 3: CSDs of two levitated particles along to identify collective modes. (a) CSDs for two particles separated by 570 µm, showing no coupled modes. **(b)** CSDs for two particles separated by 150 µm. Two modes along the x- and z-axes are coupled whilst no coupling is seen along the y-axis.

S3 Extended information for the four-particle dataset

Here we investigate the collective modes of the four levitated microparticles case shown in Fig. 2 of the manuscript. As discussed in the Supplementary Section S2, the collective modes can be found via

a CSD analysis. We show CSDs for all pairs of particles in Supplementary Figure 4, and conclude that the four levitated particles are coupled along the Paul trap x-axis (14 Hz, 25 Hz, 32 Hz and 35 Hz) due to their layout in the trap [cf. Supplementary Figure 1]. It is expected that four coupled oscillators have four coupled modes, but the nature of them is intricate and cannot be simply attributed as common or breathing modes unlike the two particle case [2, 3].

In contrast, there is only evidence of very small correlations along the y- and z-axes, and only between nearest-neighbour particles. We do not see these modes in Fig. 2 of the manuscript, and if they are real they are below our detection noise-floor. Therefore, we conclude that the motion of the four levitated microparticles is only coupled along the x-axis.

We report the signal-to-noise ratios (SNRs) of our detection for each of the four particles in Supplementary Table 1. We can see the strongest SNR is reported for particle P4, and it gradually reduces for particles further away from this. This is because we use a single laser for illumination focusing on P4. In single-particle experiments, we achieve a SNR up to 35 dB [4]. The SNR can be improved in future experiments using a higher-power illumination system which can be expanded to uniformly illuminate many particles. Alternatively, separate beams could be used for each particle, via e.g. a spatial light modulator [5, 6]. The charge to mass ratios of the four levitated particles are provided in Supplementary Table 2, from which we can observe that the four charges are different, since the masses are the same to within 10% (manufacturer specified).

	Particle 1	Particle 2	Particle 3	Particle 4
f_z SNR (dB)	3.9 ± 0.8	6.2 ± 1.1	7.4 ± 1.1	27.9 ± 1.1
f_y SNR (dB)	2.9 ± 1.1	6.5 ± 1.1	6.1 ± 1.1	19.8 ± 1.1
$x_c^{(1)}$ SNR (dB)	4.5 ± 0.8	10.9 ± 0.8	13.3 ± 0.8	15.3 ± 0.8
$x_c^{(2)}$ SNR (dB)	6.0 ± 1.1	5.0 ± 1.3	4.7 ± 1.0	12.1 ± 1.1
$x_c^{(3)}$ SNR (dB)	3.2 ± 0.8	5.1 ± 1.4	0	15.2 ± 0.9
$x_c^{(4)}$ SNR (dB)	0	3.2 ± 1.1	3.7 ± 1.0	15.6 ± 0.9

Supplementary Table 1: Detected SNRs of the four levitated particles.

	Particle 1	Particle 2	Particle 3	Particle 4
q/m (C/kg)	$(470 \pm 30) \times 10^{-5}$	$(430 \pm 10) \times 10^{-5}$	$(380 \pm 30) \times 10^{-5}$	$(310 \pm 10) \times 10^{-5}$

Supplementary Table 2: Charge to mass ratios of the four levitated particles.

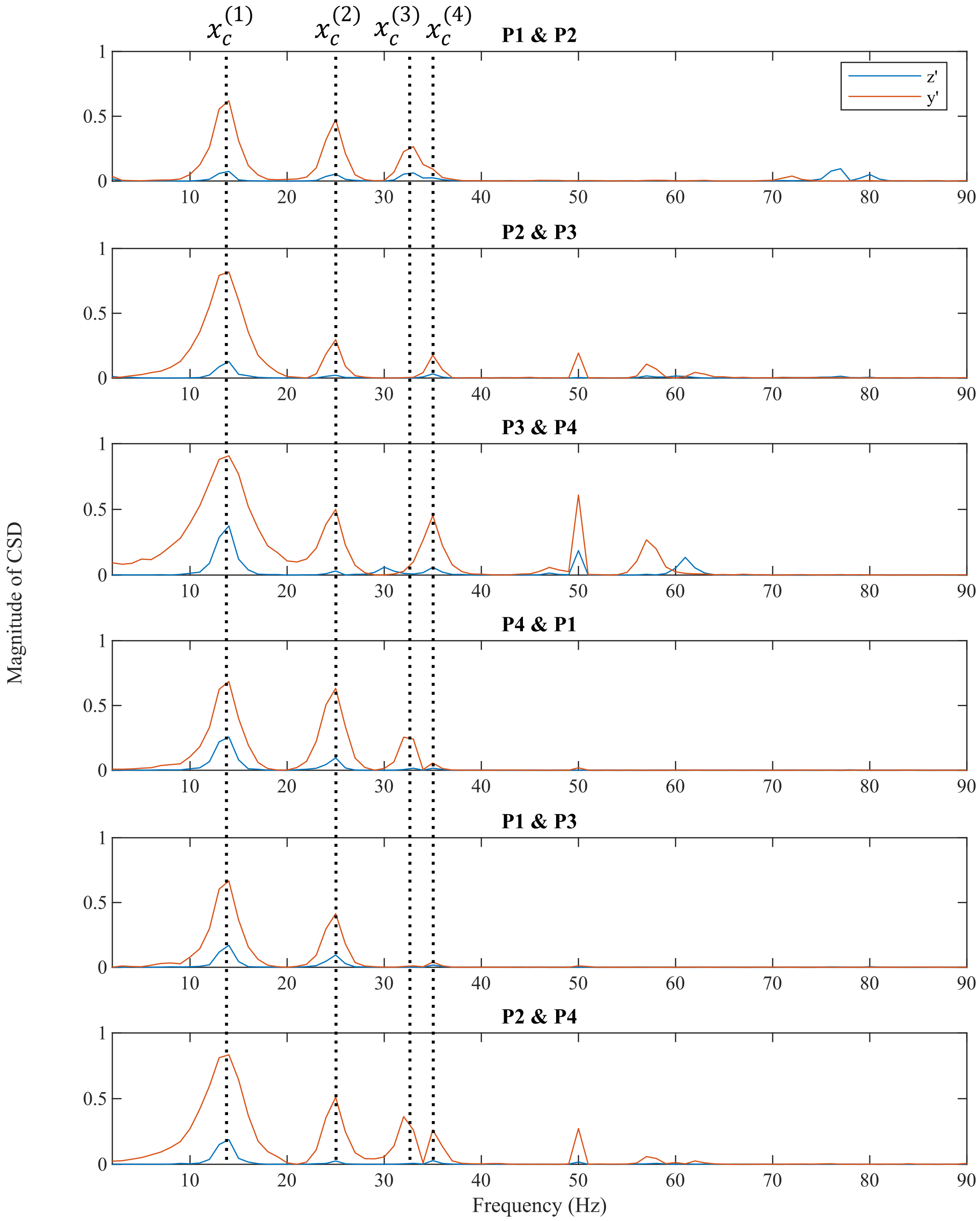

Supplementary Figure 4: CSD between the four levitated particles presented in Fig. 2 of the manuscript. The four particles are labelled P1-4. Four coupled modes along the x- axis are observed, and we see no significant coupling between the y- and z-axis modes.

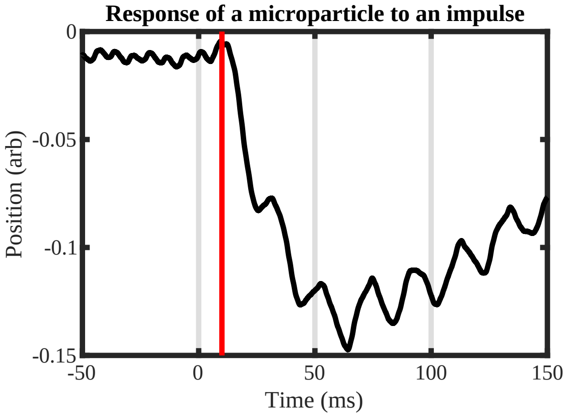

Supplementary Figure 5: Latency of the data pipeline. A levitated particle is kicked with a voltage impulse applied to the control endcap electrode at time $t = 0$. The response of the particle is recorded at the last step of the data pipeline (the output from the FPGA) and averaged over 40 realisations. The response is within 10ms, as indicated by the red line.

S4 Latency in the feedback loop

We measure the latency of the entire data pipeline by exposing a levitated particle to a voltage impulse and then monitoring the response in the data streamed from the last step in the pipeline (after the FPGA). In Supplementary Figure 5, we present the result averaged over 40 realisations. We find that the response is within 10 ms.

Our data-pipeline is sub-optimal, since data is transferred from the event based camera via a PC to the FPGA. In the future we aim to use an FPGA directly connected to the camera hardware. In such a case, the primary limitation would be the sensor latency (200 µs) and the communication bandwidth between the camera hardware and a multi-channel DAC. However, in our current system:

- Latency from the changes in light intensity at each pixel to event output: typical 200 µs from the detector manual.
- Data Transfer to Computer: The camera uses USB 3.0 at a maximum data-rate of 4 GBit/s – this will never be the limiting case because it's much larger than the data transfer at maximum event-rate. USB 3.0 has a latency of about 30 µs.
- The EBC software then tracks the objects – latency in this step is very hard to evaluate, since the process is proprietary.
- Python code processes the tracking data for it to be sent to the FPGA, this is simple and can be considered negligible.

- The data is transferred via Ethernet and a network switch to our collection of FPGAs, which has a latency of about 300 µs.
- Data Processing on FPGA: Each FPGA computes velocity, applies gain and a phase shift, and outputs a feedback signal. Since our FPGA runs at 124 Msps, it is reasonable to say the latency is negligible compared to the data transfer delays.

The delay of applied control signal has two effects (following Ref. [7]). Firstly, it deterministically shifts the phase between the mechanical system and the feedback signal, leading to oscillatory behaviour in the correlations and thus a transition between heating and cooling. Secondly, it causes stochastic dephasing of the mechanical motion relative to the feedback signal as the delay becomes large, which reduces the strength of the correlations.

From the experimental results of Ref. [7], correlation functions of the microparticle centre-of-mass motion as a function of delay remain high for short delays (on the order of a few oscillation periods) when the dynamics are underdamped (as in our system). Since our levitated oscillators have oscillation frequencies below 100 Hz, the latency of 10 ms corresponds to a single period of delay, and has little consequence for our cooling protocol.

S5 Power consumption of the event based camera

From the neuromorphic sensor manual (PPS3MVCD) the static power consumption of the EBC is 26 mW, plus a dynamic power consumption based on sensor activity. In our experiments the event rate is about 500 kevt/s, leading to a power consumption of about 27.6 mW. This is very low compared to a CMOS camera (Thorlabs CS165MU/M, 1.17 W Max @ 34.8 fps Full Sensor ROI) or a high-speed camera (iX Cameras i-SPEED 230, 17 W at 2500 fps).

S6 Bath temperatures T_0 for all particles

Due to voltage noise from the amplifiers driving our Paul trap, the equilibrium temperature of our particles without cooling ranges from approximately $T_0 = 400 - 1500\,\mathrm{K}$. This temperature varies depending on their charge and spatial location within the trap. Here we model the electrical noise as a white noise bath and assume the particles are trapped in a quadratic potential. Under the assumption that the equipartition theorem holds, the different bath temperatures used in the main text figures are listed in Supplementary Table 3. In Fig. 4(c), P2 is located closest to the trap center and therefore experiences the least electric field noise, resulting in the lowest bath temperature. In contrast, P1 and P3 are farther from the trap center, and their bath temperatures are approximately an order of magnitude higher than

that of P2. A similar trend can be observed in Fig. 4(b), where P1, being farther from the trap center, also exhibits a higher temperature than P2.

	Fig3(b)	Fig3(c)	Fig3(d)	Fig4(a)	Fig4(b)	Fig4(c)
T_0 (K)	900 ± 300	1500 ± 100	400 ± 50	500 ± 70	P1:2300 ± 300<br>P2:600 ± 100	P1:3300 ± 600<br>P2:400 ± 50<br>P3:4300 ± 800

Supplementary Table 3: Different equilibrium temperature T_0 values in the main text figures.

S7 Cooling limit and the noise squashing

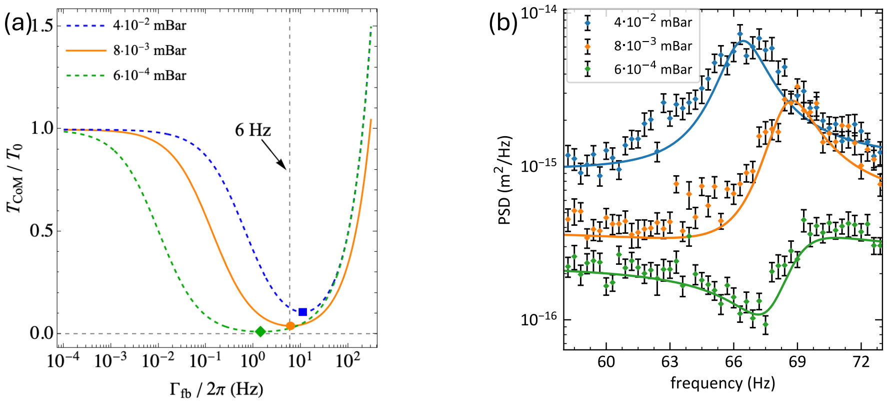

Supplementary Figure 6: Cooling limit and the noise squashing. (a) The cooled temperature of the system T_{CoM} against the feedback rate Γ_{fb} at different pressures given by equation (2) in the main manuscript. Here the dots are the optimal feedback rates to reach the corresponding minimum cooling temperature, computed from Supplementary Equations (1) and (2). **(b)** The experimental PSDs of particle's measured motion fitted by Supplementary Equations (3), showing the change from unsquashed (blue) to squashed (green) as the pressure reduces. Here we set feedback rate $\Gamma_{\mathrm{fb}}/2\pi \approx 6$ Hz. The experimental error-bars in the figure are derived by taking 15 repeat experiments at each set of parameters to calculate a mean and standard deviation.

At a certain pressure (which determines the gas damping rate Γ_0), to achieve maximal cooling the optimal feedback rate Γ_{fb} can be obtained from equation (2) in the main manuscript by taking $\partial T_{\mathrm{CoM}}/\partial \Gamma_{\mathrm{fb}} = 0$ [8], which gives

$$T_{\mathrm{CoM}}^{\mathrm{opt}} = \frac{-m\Gamma_0\omega_{\mathrm{z}}^2 S_{\mathrm{nn}} + \sqrt{m\Gamma_0\omega_{\mathrm{z}}^2 S_{\mathrm{nn}}(2k_{\mathrm{B}}T_0 + m\Gamma_0\omega_{\mathrm{z}}^2 S_{\mathrm{nn}})}}{k_{\mathrm{B}}} \qquad \text{(Supplementary Equation 1)}$$

with the optimal feedback rate

$$\Gamma_{\mathrm{fb}}^{\mathrm{opt}} = \frac{1}{\cos(\phi)}\left(-\Gamma_0 + \frac{\sqrt{m\Gamma_0\omega_{\mathrm{z}}^2 S_{\mathrm{nn}}(2k_{\mathrm{B}}T_0 + m\Gamma_0\omega_{\mathrm{z}}^2 S_{\mathrm{nn}})}}{m\omega_{\mathrm{z}}^2 S_{\mathrm{nn}}}\right) \qquad \text{(Supplementary Equation 2)}$$

Note that $T_{\rm CoM}^{\rm opt}$ is ϕ-independent.

The relation between the feedback rate $\Gamma_{\rm fb}$ and the cooling temperature $T_{\rm CoM}$ is shown in Supplementary Figure 6(a), where we plot equation (2) in the main manuscript at three different pressures. The dots in the plot are the optimal feedback rates and the optimal temperatures corresponding to the respective pressure, computed from Supplementary Equations (1) and (2). As we can see, the optimal feedback rate gets smaller as the pressure goes down.

At a certain pressure, a larger feedback rate $\Gamma_{\rm fb}$ results in a lower system temperature $T_{\rm CoM}$ (hence a better cooling), until it pass the the optimal value in which case the feedback signal instead heats up the system. This is because the increasing amplitude of the feedback signal also amplifies the noise fed back to the particle, preventing further cooling. The corresponding PSD also appears inverted below the noise floor, a phenomenon known as the noise squashing [9]. In Supplementary Figure 6(b) we demonstrate this by setting the feedback rate $\Gamma_{\rm fb}/2\pi = 6$ Hz and reducing the pressure. Correspondingly, the PSDs of the measured motion changes from unsquashed (blue line, $\Gamma_{\rm fb} < \Gamma_{\rm fb}^{\rm opt}$), to mixed (orange line, $\Gamma_{\rm fb} \approx \Gamma_{\rm fb}^{\rm opt}$), and finally to squashed (green line, $\Gamma_{\rm fb} > \Gamma_{\rm fb}^{\rm opt}$). Furthermore, the squashed shape is asymmetrical due to the delay in the signal [10]. This makes a small change to the fitting PSD function, as shown in the following:

Suppose we consider a time delay in the feedback signal $v(t) \to v(t-\tau)$, the equation of motion reads

$$\ddot{z} + \Gamma_{\rm g}\dot{z} + \omega_0^2 z = \frac{\sigma}{m}\eta(t) - \Gamma_{\rm fb}(v(t-\tau) + \dot{\xi}(t)).$$

Take the Fourier transform with $z(\omega) = \mathcal{F}[z(t)] = \int_{-\infty}^{\infty} z(t)e^{-i\omega t}{\rm d}t$ and $\mathcal{F}[z(t-\tau)] = e^{-i\omega\tau}\mathcal{F}[z(t)]$, we get the particle's detected motion in the frequency domain

$$\begin{aligned}\chi'(\omega) + F(\xi(t)) =& \frac{\sigma/m}{(\omega_0^2 - \omega^2 + \omega\Gamma_{\rm fb}\sin(\omega\tau)) + i\omega(\Gamma_{\rm g} + \cos(\omega\tau)\Gamma_{\rm fb})}F(\eta(t)) \\ &+ \frac{\omega_0^2 - \omega^2 + i\omega\Gamma_{\rm g} + i\omega\Gamma_{\rm fb}(e^{-i\omega\tau} - 1)}{(\omega_0^2 - \omega^2 + \omega\Gamma_{\rm fb}\sin(\omega\tau)) + i\omega(\Gamma_{\rm g} + \cos(\omega\tau)\Gamma_{\rm fb})}F(\xi(t)),\end{aligned}$$

We can calculate the PSD in this situation by taking $S_{\rm z}(\omega) = {\rm IE}(|\chi'(\omega)^2|)$ and assume that two noises $F(\eta(t))$ and $F(\xi(t))$ are uncorrelated, which gives the PSD of the measured position in the in-loop detector:

$$\begin{aligned}S_{\rm IL}(\omega) =& \frac{\sigma^2/m^2}{(\omega_0^2 - \omega^2 + \omega\Gamma_{\rm fb}\sin(\omega\tau))^2 + (\Gamma_{\rm g} + \cos(\omega\tau)\Gamma_{\rm fb})^2\omega^2} \\ &+ \frac{(\omega_0^2 - \omega^2 + \omega\Gamma_{\rm fb}\sin(\omega\tau))^2 + \omega^2(\Gamma_{\rm g} + \Gamma_{\rm fb}(\cos(\omega\tau) - 1))^2}{(\omega_0^2 - \omega^2 + \omega\Gamma_{\rm fb}\sin(\omega\tau))^2 + (\Gamma_{\rm g} + \cos(\omega\tau)\Gamma_{\rm fb})^2\omega^2}S_{\rm nn}.\end{aligned}$$

(Supplementary Equation 3)

This signal delay τ can cause a small asymmetry that is visible in the squashed PSD from in-loop detection. In this case, the experimental PSD of particle's measured motion can be correctly fitted by Supplementary Equations (3), as shown in Supplementary Figure 6(b).

S8 Scalability of multi-mode cooling

Here we discuss the fundamental limit on the number of modes N we can cool with the technique presented in our manuscript. We assume N is bounded by the bandwidth of our detection $F \approx 500\,\text{Hz}$ and the minimum resolvable linewidth Δf, such that $N \approx \frac{F}{\Delta f}$. Then, to estimate the number of modes, we need to find the minimum linewidth Δf.

The contribution to this limit from the experimental setup comes mainly from filtering. Considering the time duration for signal demodulation, there is a bandwidth limit for filters to pick out certain frequency signal which will set a limit to Δf. A PLL has a very narrow filter bandwidth, for example a Zurich Instruments HF2LI has a minimum bandwidth of 83 µHz and can filter 6 peaks at once. Therefore, with PLL it is possible to pick up feedback signal at mHz resolution.

On the other hand, since we use cold damping to cool the particles, the particles inevitably experience an additional damping rate, which broadens the linewidth and makes the modes harder to distinguish. According to Eq. (2) in the manuscript, by solving $\partial T_{\text{CoM}}/\partial \mathit{\Gamma}_{\text{fb}} = 0$, we can obtain an optimal $\mathit{\Gamma}_{\text{fb}}$ at a given pressure (See Supplementary Equations (2)). This optimal $\mathit{\Gamma}_{\text{fb}}$ would be the dominant factor in determining the scaling number, whose value reduces as pressure goes down.

If we take the above argument as a rule of thumb, we can estimate the number of modes as follows. Suppose we start cooling at 10^{-4} mbar in experiment, at which the particle's linewidth is about $\mathit{\Gamma}_0/2\pi \approx 0.002\,\text{Hz}$, the optimal feedback rate is $\mathit{\Gamma}_{\text{fb}}/2\pi \approx 1.8\,\text{Hz}$. This limits the capacity of our setup to be $N \approx 270$ modes, which can be improved at high vacuum.

References

1. Penny, W. D. Signal processing course. Citeseer, 2000.
2. Penny, T., Pontin, A. & Barker, P. Sympathetic cooling and squeezing of two colevitated nanoparticles. *Physical Review Research* **5**. 013070 (2023).
3. Watson, D. Normal modes for N identical particles: A study of the evolution of collective behavior from few-body to many-body. *Annals of Physics* **419**. 168219 (2020).
4. Ren, Y. et al. Event-based imaging of levitated microparticles. *Appl. Phys. Lett.* **121** (2022).
5. Obata, K. et al. Multi-focus two-photon polymerization technique based on individually controlled phase modulation. *Optics express* **18**. 17193–17200 (2010).
6. Rieser, J. et al. Tunable light-induced dipole-dipole interaction between optically levitated nanoparticles. *Science* **377**. 987–990 (2022).
7. Debiossac, M. et al. Thermodynamics of continuous non-Markovian feedback control. *Nat. Commun.* **11**. 1360 (2020).
8. Melo, B. et al. Vacuum levitation and motion control on chip. *Nat. Nanotechnol.* **19**. 1270–1276 (2024).
9. Poggio, M. et al. Feedback Cooling of a Cantilever's Fundamental Mode below 5 mK. *Phys. Rev. Lett.* **99**. 017201 (2007).

10. Conangla, G. P. et al. Optimal Feedback Cooling of a Charged Levitated Nanoparticle with Adaptive Control. *Phys. Rev. Lett.* **122**. 223602 (2019).
